# Study on the spatialization of anthropogenic carbon emissions in China based on SVR-ZSSR

**DOI:** 10.1038/s41598-023-28462-x

**Published:** 2023-02-02

**Authors:** Minghao Liu, Liai Qi, Haiyan Chen, Xiaolin Luo, Xiaobo Zhu, Chun Chen

**Affiliations:** 1 College of Computer Science and Technology, Chongqing University of Posts and Telecommunications, Chongqing, 400065 China; 2 Spatial Information Research Center, Chongqing University of Posts and Telecommunications, Chongqing, 400065 China; 3 College of Architecture and Urban Planning/Eco-Habitat and Green Transportation Research Center, Chongqing Jiaotong University, Chongqing, 400074 China

**Keywords:** Computer science, Climate and Earth system modelling, Energy and behaviour

## Abstract

The spatialization of anthropogenic carbon emissions is of great significance for achieving the goal of "carbon peaking and neutrality" and promoting the development of carbon trading market. The SVR-ZSSR spatialization model is proposed to solve the problems of "insufficient local feature learning" and "scale dependence of driving factors" existing in the single model, and this model is applied to the study of the spatialization of carbon emissions in China in this paper. The results show that: (1) the simulation results of our proposed model show the distribution characteristics of "high in the East and low in the west"; On the micro-scale, the high carbon emission areas in the simulation results are all concentrated in the built-up land, while the carbon emission in the surrounding areas of the city is significantly lower than that in the center, which is similar to the spatial distribution trend of carbon emission in the existing database. (2) Compared with the results of SVR, the results of our proposed model increase the carbon emission ratio of built-up land in each province by 15.9% on average; Compared with the ODIAC database and SVR model, the carbon emission ratio of built-up land in Gansu Province, Qinghai Province, and other low-carbon emission areas has increased by about 25%.

## Introduction

Global climate change has attracted international attention because of its great threat to the earth’s environment and human survival. Available evidence suggests that increased human activity will accelerate the process of global warming^1^. Therefore, all countries in the world need to take practical actions to reduce the emission of greenhouse gases such as carbon dioxide and delay the process of global warming, to meet the challenges brought by climate change. In 2016, 175 countries signed *the Paris Agreement*, which set a temperature control target of 2 °C for each signatory. Those countries must achieve a balance between anthropogenic emissions by sources and removals by sinks of greenhouse gases by 2050. As of October 2020, there were 127 carbon-neutral pledged countries around the world, and about half of global greenhouse gas emissions come from these countries^2^. The average annual growth rate of China's carbon emissions has been around 10% since 2000, which accounts for about 29% of the total global carbon emissions^3^. China is currently one of the countries with the largest carbon emissions in the world. China made a commitment to the world at the UN General Assembly in September 2020 that it would strive to peak its carbon dioxide emissions by 2030 and achieve carbon neutrality by 2060 (referred to as the "dual carbon" goal).

The realization of the "dual carbon" goal includes three steps: estimating carbon emissions, reducing carbon emissions, and offsetting carbon emissions. In the past two decades, researchers from all walks of life in China have made many innovations in carbon emission estimation methods, and have deeply studied the time curve of carbon emissions under various scenarios. However, most studies focus on the temporal changes of carbon emissions, and the spatial scales of their studies are often based on macro-scales such as countries, provinces, and cities, and detailed studies on the spatial scale are still lacking. The improvement of the carbon trading market, the distribution of carbon emissions responsibilities, the collection of carbon taxes, and the regulation of carbon emissions within cities and between regions all need to rely on carbon emissions distribution data with higher spatial and temporal resolution.

Data spatialization can transform province-based statistics into spatially distributed data with a higher spatial resolution (e.g., a 1 km × 1 km grid) by using models and associated data. Spatialization research on data spatialization for many years has mainly focused on the simulation of population and GDP. Research on the spatialization of carbon emissions has only increased in recent years. Nighttime lights are a type of remote sensing data. Sensors on satellites can detect information such as the lights and fires of the earth at night, so this data can be a good indicator of human activities. Many scholars have used nighttime lights data in carbon emissions spatialization research^4,5^. Although this method is widely used, it still has some limitations. For example, the relationship between light data and carbon emissions is very complex^6^, and light data tends to be highly biased in less developed areas such as rural areas^7^. Therefore, using only nighttime lights data may lead to large errors. In recent years, multi-source data has been introduced into the study of carbon emissions to improve its spatial accuracy^8,9^. Combined with multi-source data, the problem of saturation overflow and missing of nighttime lights data is alleviated to a certain extent. Besides, the introduction of point source data is also an important method to improve the accuracy of spatial resolution^10,11^. However, it is difficult to obtain point data, which often increases the difficulty of research. Machine learning methods such as Random-Forests and Bayesian-Algorithms have been widely used in the study of carbon emissions spatialization. But such models only treat the study units as independent and unified, ignoring the heterogeneity of the spatial distribution of carbon emissions data. To lift this limitation, local models are introduced into spatialization studies.

Common local models include the geographically weighted regression (GWR) model and the super-resolution (SR) model. GWR can spatialize statistical data. However, its spatializing results are often limited by the spatial scale of the driving factors. When using the driving factors of 1 km spatial resolution, the model also produces results of only 1 km and cannot be a higher spatial scale (e.g., 500 m). This means that as the spatial resolution of the study increases, driving factors with higher spatial resolution are required. Super-resolution models have no higher requirements on the spatial resolution of the drivers. SR has been extensively studied in computer vision. The main problem solved in this field is the conversion from low-resolution images to high-resolution images. The model can learn the local features of the image, so as to complete the more accurate restoration of the enlarged image. Research by Vandal et al.^12^ demonstrated that there is a certain correspondence between super-resolution of a single image and downscaling techniques for geospatial information. The mapping between low-resolution and high-resolution of spatial information can also be achieved by super-resolution models. Zong et al.^13^ combined the Super-Resolution Convolutional Neural Network model (SRCNN) to realize the population spatialization of Shanghai. The experimental results show that this method can obtain more accurate population spatial distribution information. Although the SRCNN model improved the effect by introducing a deep learning model, it requires complete training data and has higher requirements for label data (high-resolution images). However, in the field of carbon emissions, there is no one-to-one correspondence between high and low resolution data, so the SRCNN model is not suitable for the study of carbon emissions spatialization. Glasner et al.^14^ believed that there were multiple repeated image patches inside natural images, and these image patches would still appear repeatedly at various scales. Zontak and Irani^15^ found that partial information in images can provide sufficient prior knowledge for image restoration, and applying this prior knowledge tends to achieve better results than using external image training. Shocher et al.^16^ proposed an unsupervised neural network-based super-resolution method called zero-shot super-resolution (ZSSR). The method does not require pre-training, nor does it require pairs of high and low resolution images corresponding to each other. The model improves the resolution of upscaled images by learning the internal details of low-resolution images. The ZSSR model that can learn local features is proposed to be introduced into the traditional regression model to learn the spatial distribution characteristics of carbon emissions and improve the spatial resolution of carbon emissions spatialization in this paper.

In summary, in view of the shortage of quantitative research on carbon emissions on fine spatial scales, and the problem that a single model cannot fully learn the spatial distribution characteristics of carbon emissions, this paper intends to combine the global model with the local model to construct a hybrid model suitable for carbon emissions spatialization. The model can also reduce the dependence on the spatial scale of driving factors to obtain better results of carbon emission distribution at high spatial resolution. Based on the calculation method of this study, the carbon emissions in this study refer to carbon dioxide emissions. First, compare and select multiple regression models, and select the model with the best effect. Based on this model, the coarse-grained spatialization results of the national 1 km carbon emissions are generated; then, the coarse-grained spatial results are input into the ZSSR model as "low-resolution images" to generate the fine-grained spatialization results of the national 500-m carbon emissions.

## Results

### "Coarse" scale spatialization results

Linear Regression is the most classic regression model, and it is also the simplest regression model, which can well represent the linear relationship between the independent variable and the dependent variable. Bayesian Linear Regression is adaptive to the data and prevents overfitting. ElasticNet Regression shows good performance when dealing with parameters that are correlated. Support Vector Regression is simple to implement and has excellent generalization ability and prediction accuracy. These models have their own characteristics, and they are widely used in various fields. So, these four models were used in this study. The results parameters of each regression model are shown in Table 1. The results were generated using the 2010 provincial carbon emission statistics as the training data, the 2005 provincial carbon emission statistics as the test data and the decision coefficient (R^2^), mean absolute error (MAE), and root mean square error (RMSE) as the evaluation indicators of the model. Compared with various parameters, Support Vector Regression (SVR) achieved good results in all aspects. Therefore, the SVR model was selected as the basis of the subsequent composite model.

**Table 1:** SVR Regression results.

	R^2^	MAE	RMSE
SVR	0.72	1469.00	1782.92
Bayesian Linear Regression	0.63	1769.48	2053.10
Linear Regression	0.65	1615.21	2158.20
ElasticNet Regression	0.65	1673.63	1988.81

**Figure 1:**
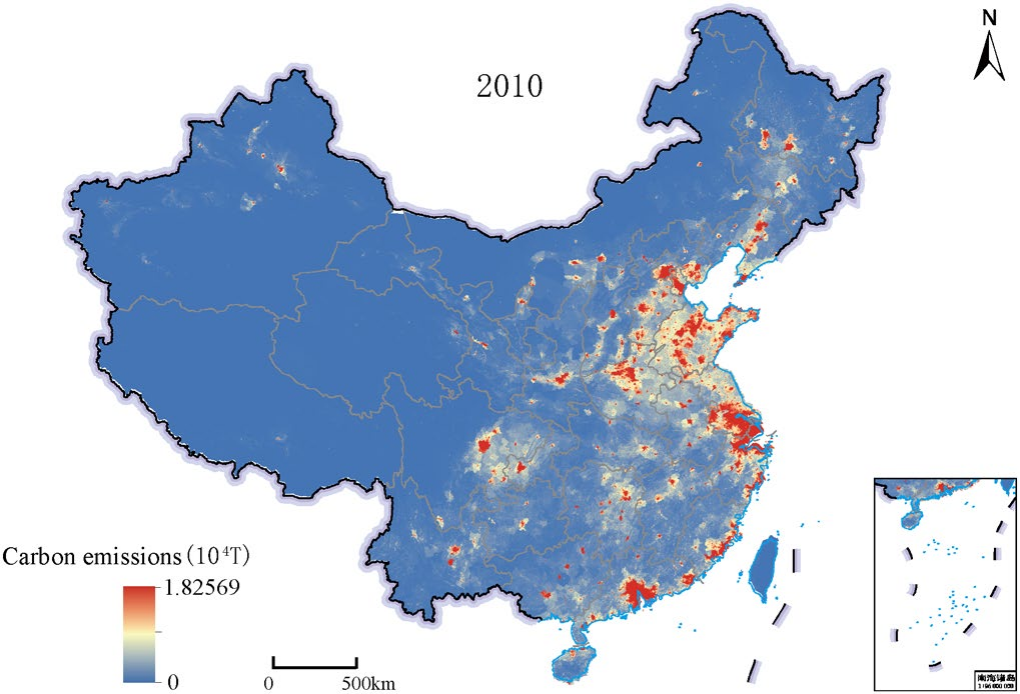
Results of 1 km spatialization of Carbon Emissions in China in 2010 based on the SVR model. This map is based on the standard map No. GS(2019) 1823 downloaded from the Ministry of Natural Resources Standard Map Service website. The base map is unchanged.

The 1 km spatial map of China's carbon emissions in 2010 was obtained by taking the 1 km spatial scale driving factor data as the input data of the SVR model, as shown in Fig. 1. SVR model had well learned the global characteristics of carbon emissions and refined the spatial scale of carbon emissions from a provincial scale to a 1 km spatial resolution scale, which can be used as the "low-resolution image" of the ZSSR model.

### "Fine" scale spatialization results

**Figure 2:**
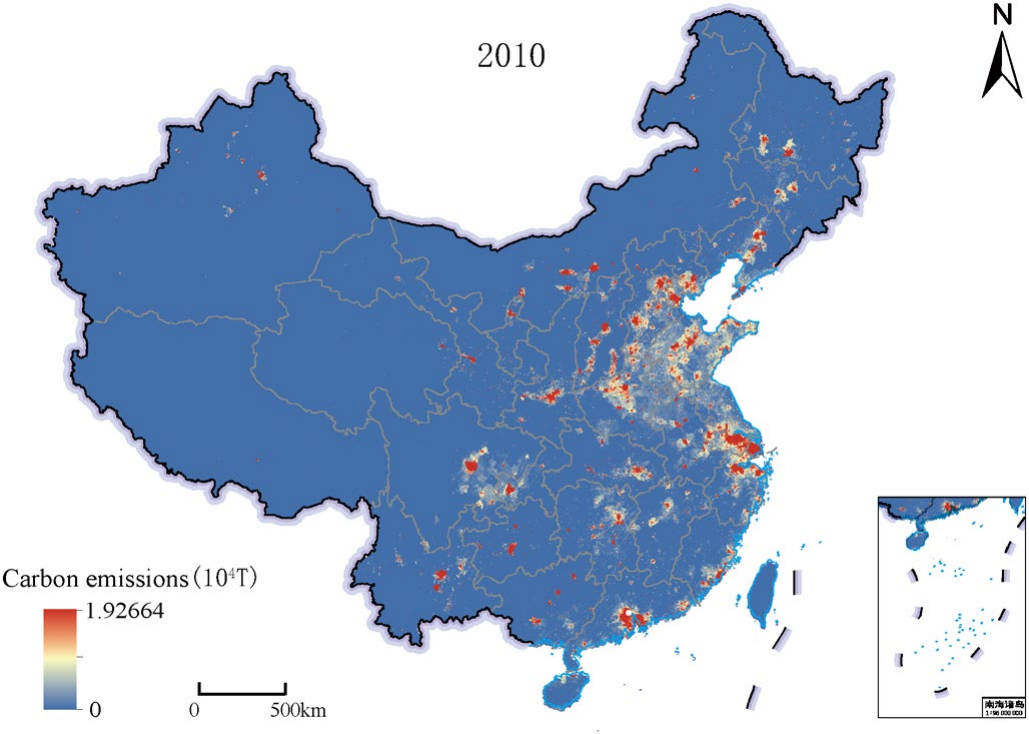
Results of 500 m spatialization of carbon emissions in China in 2010 based on the SVR-ZSSR model. This map is based on the standard map No. GS(2019) 1823 downloaded from the Ministry of Natural Resources Standard Map Service website. The base map is unchanged.

Taking the 1 km carbon emissions spatialization results as input, the 500 m national carbon emissions spatialization results were obtained through the SVR-ZSSR model's training and correction of provincial carbon emissions data, as shown in Fig. 2. High carbon emissions areas were mostly distributed in coastal cities and economically developed provincial capital cities in China. The overall carbon emissions trend of China was high in the East and low in the west, which was similar to the provincial carbon emissions distribution pattern, as shown in Fig. 7.

#### Analysis of representative provincial and municipal results

**Figure 3:**
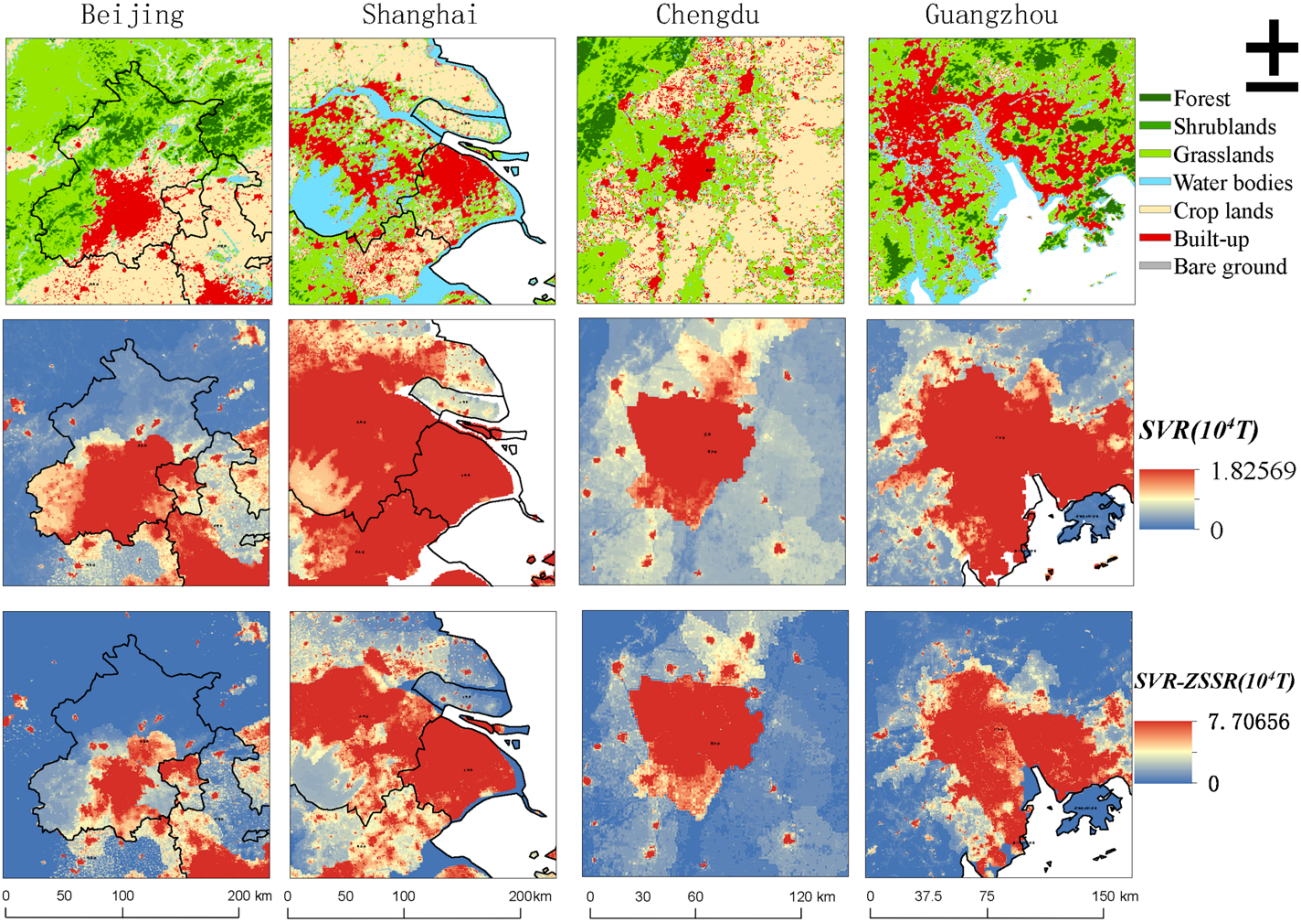
Comparison of SVR model and SVR-ZSSR model results. The figs were generated using the ArcGIS Desktop (ESRI, Inc, Version 10.7, https://desktop.arcgis.com/zh-cn/).

**Figure 4:**
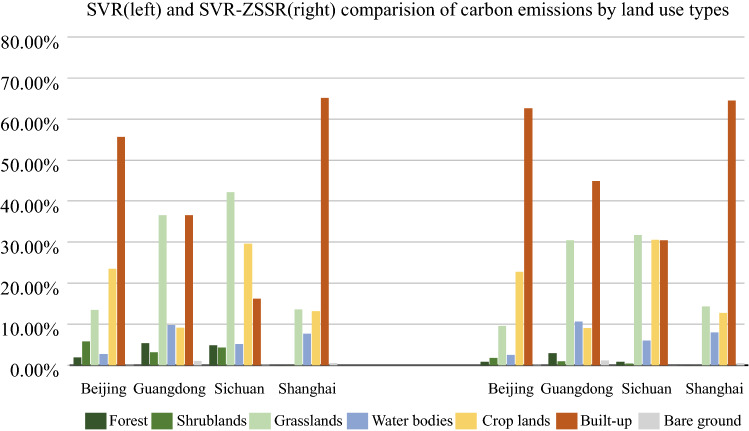
Comparison of carbon emission ratios of different land use types between SVR model and SVR-ZSSR model.

Some representative regions like Beijing, Shanghai, Chengdu (Sichuan Province), and Guangzhou (Guangdong Province) were selected for qualitative analysis on the 500 m spatial scale in this study. These four regions have rapid social and economic development, concentrated population, and complete land use types, and they are all representative central cities in China. The comparison of the results of the SVR model and SVR-ZSSR was shown in Fig. 3, and the unit was 10^4^ T. It can be found that the carbon emissions of the four regions were mainly concentrated in the built-up land, but the latter was more concentrated. The carbon emissions of the non-built-up land around the built-up land in the SVR-ZSSR model were lower than the SVR model's result, which can clearly distinguish the carbon emissions of the built-up land from those of other land types. Figure 4 showed the proportions of carbon emissions of different land use types based on the SVR model and SVR-ZSSR model. It was shown that the SVR-ZSSR model did a good job of concentrating carbon emissions on built-up land. For example, the proportion of grasslands carbon emissions in Guangdong Province was 36.53%, and the proportion of built-up land carbon emissions was 36.60% under the SVR model, while the proportion of built-up land carbon emissions in Guangdong Province can increase to 44.88% by using the SVR-ZSSR model. Under the SVR model, the proportion of grassland carbon emissions in Sichuan Province was 42.11%, the proportion of croplands’ carbon emissions was 29.56%, and the proportion of built-up land carbon emissions was only 16.18%. While using the SVR-ZSSR model can increase the proportion of built-up land carbon emissions to 30.52%.

#### Comparison of the spatial distribution of provinces in China

**Figure 5:**
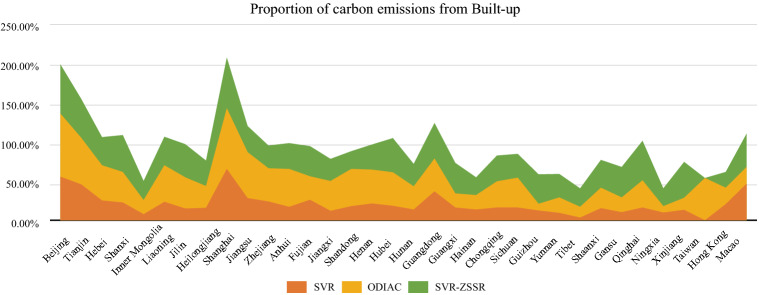
Comparison of SVR model and SVR-ZSSR model in the proportion of carbon emissions in built-up land in each province.

In addition to typical cities, this study also made statistics on the carbon emissions ratio of built-up land in 34 provinces of China in SVR, SVR-ZSSR, and Open-source Data Inventory for Anthropogenic CO_2_ (ODIAC) databases (the data of two models in Taiwan Province was shown as 0). It can be seen from Fig. 5 that the carbon emissions ratio of built-up land in the SVR-ZSSR model was higher than that in the SVR model except in Hong Kong and Macao, and the carbon emissions ratio of built-up land in each province had increased by 15.9%. In addition, compared with the ODIAC carbon emissions database, SVR-ZSSR's carbon emissions ratio of built-up land in low-carbon emissions areas such as Gansu Province, Qinghai Province, Guizhou Province, Yunnan Province, and Xinjiang Uygur Autonomous Region has increased, and the carbon emissions ratio of built-up land in Guangxi Province, Guizhou Province, Xinjiang Uygur Autonomous Region and Gansu Province has increased by more than 20%. Compared with the SVR model, the SVR-ZSSR also increased the carbon emissions of built-up land in these areas by more than 25%, which proved that the SVR-ZSSR model can significantly improve the spatialization accuracy of low-carbon emission areas. The above results all show that the SVR-ZSSR two-stage model can more accurately reflect the spatial distribution of carbon emissions in China, and its effect is more significant in low-carbon emission areas.

#### Comparison of multiple carbon emissions data sets

**Figure 6:**
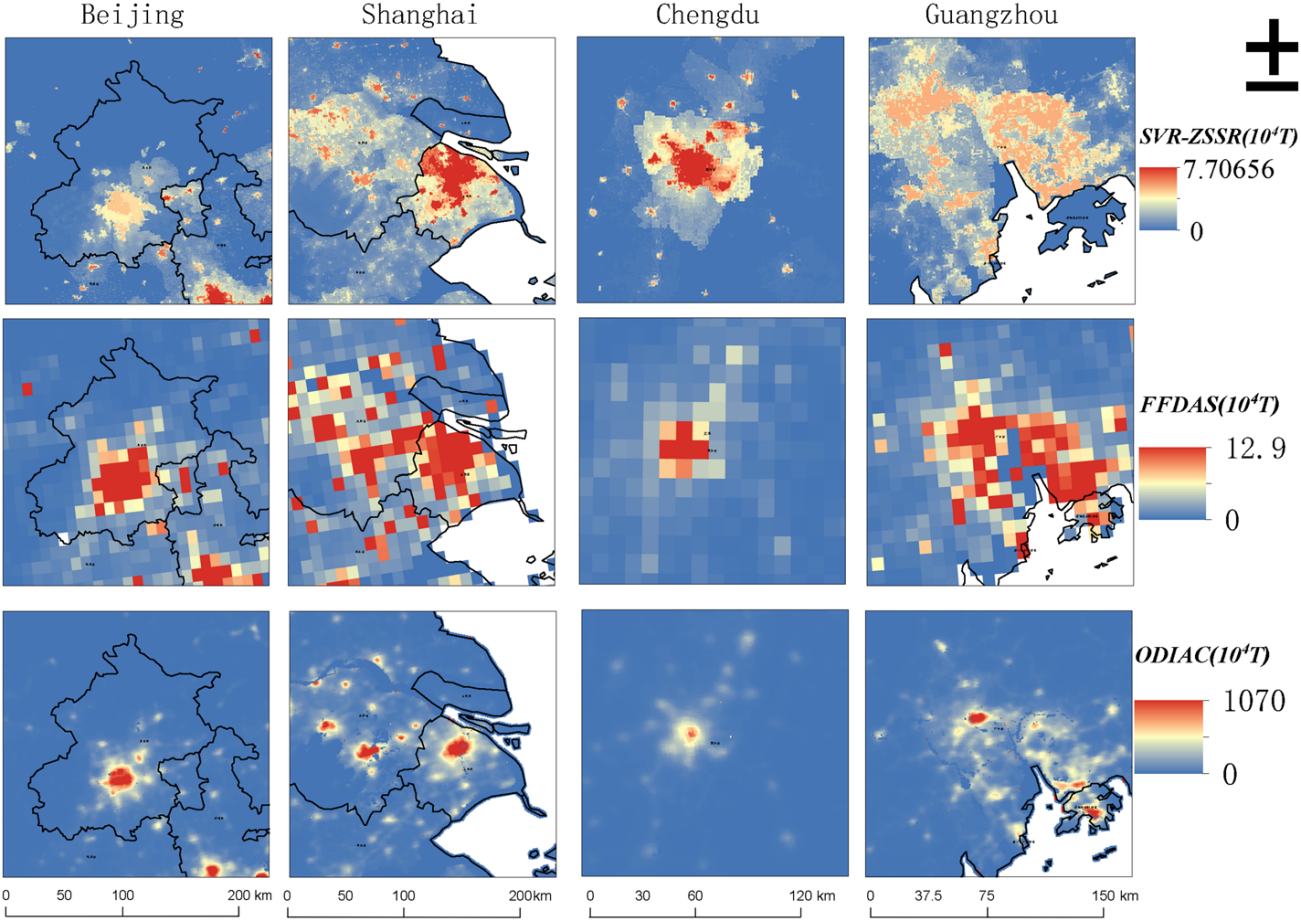
Comparison of SVR-ZSSR model results, FFDAS database, and ODIAC database. The figs were generated using the ArcGIS Desktop (ESRI, Inc, Version 10.7, https://desktop.arcgis.com/zh-cn/).

The comparison of the SVR-ZSSR model’s results with the Fossil Fuel Data Assimilation System (FFDAS) carbon emissions database and ODIAC carbon emissions database is shown in Fig. 6. The SVR-ZSSR model greatly improved the spatial resolution of the FFDAS database in particular. In addition, the spatialization result of carbon emissions in this study was 500 m spatial resolution. It proves that the SVR-ZSSR model can improve the spatial resolution while the spatial distribution trend of carbon emissions was similar to the existing database. And this model can estimate carbon emissions in a smaller spatial range.

## Conclusion

Spatialization of economic and social parameters such as carbon emissions is a hot research topic in spatial information disciplines. The introduction of deep learning and other models has greatly improved the research accuracy in this field. This study introduced the super-resolution model ZSSR in the field of image research and combined this model with the regression model to build an SVR-ZSSR two-stage model suitable for carbon emissions. The model can combine multi-source data for spatial analysis of carbon emissions, and can learn global and local carbon emissions characteristics. The experimental results show that, compared with the SVR regression model, the SVR-ZSSR model can increase the spatial resolution of carbon emissions to 500 m or even higher, and can better reflect the spatial distribution of carbon emissions at a very high spatial resolution. The model has increased the proportion of carbon emissions from built-up land in China's provinces and cities by an average of 15.9%, and the proportion in low-carbon emission areas can even increase by more than 25%.

The spatial distribution of carbon emissions reflects the overall distribution characteristics of "high in the East and low in the west", and the carbon emissions of coastal cities and economically developed cities are higher. Within the city, carbon emissions are mainly distributed in the built-up land in the city center. The results of this model are consistent with the actual situation from both macro and micro perspectives.

## Discussion

Super Resolution (SR) is the process of recovering a high-resolution (HR) image from a given low-resolution (LR) image, ZSSR is a self-supervised method, but it requires a picture for self-learning "Low-res image" of internal information. The SVR-ZSSR two-stage model is an overall model with two stages mutually supporting and conditioned on each other. The first-stage model provides a "low-resolution image" for the second-stage ZSSR model, which is a prerequisite for the second-stage to function, and its main task is to improve statistical accuracy. The second stage is a continuation of the first stage, and mainly completes the task of improving spatial accuracy. Therefore, the accuracy of the first stage method is mainly verified by statistical data evaluation indicators. The accuracy of the second stage method is mainly verified by the spatial accuracy verification method. The two-stage coupled model is well used in the spatialization of carbon emissions in this study.

However, there is still something to improve in this model. The results of this model are similar to the spatial distribution trend of the existing carbon emissions database. Due to the lack of accurate points data, there are still some differences in their numerical values. In the follow-up study, we will consider how to effectively use the points sources data in the public data to improve the accuracy of the spatial distribution of carbon emissions. In addition, the optimization of the "low-resolution image" method and the selection of driving factors have a great influence on the test results. During the construction of the "low-resolution image", this study optimized the pre-model selection and considered the multi-source data including nighttime lights data, population, and GDP in the selection of driving factors. In the follow-up study, better methods and more human activity intensity point data closely related to the spatial and temporal distribution of carbon emissions, such as power plants and dynamic traffic, can be selected in the first stage to improve the simulation accuracy of the model.

## Materials and methods

### Data and preprocessing

The data used in this study include carbon emissions data, driving factor data, and validation data. The basic information of the data source is shown in Table 2. The carbon emissions data mainly include the statistical data on energy consumption of various provinces and cities. The driving factor data affecting carbon emissions mainly include nighttime lights data, population grid data, GDP grid data. Carbon emissions verification data includes FFDAS carbon emissions data, ODIAC carbon emissions data and land-use data.

**Table 2:** Data description.

Data	Data description	Particular year	Data sources
Energy consumption	Physical consumption and total energy consumption of coal, coke, crude oil, gasoline, kerosene, diesel, fuel oil, and natural gas in 30 provinces and cities in China	2005, 2010	China Energy Statistics Yearbook
Global NPP-VIIRS nighttime lights data	Annual nighttime lights data with a spatial resolution of 500 m	2005, 2010	Harvard database (https://dataverse.harvard.edu/)
Spatial distribution of GDP	Spatial distribution data of China's GDP with a spatial resolution of 1 km	2005, 2010	Resources and environment science and data center of Chinese Academy of Sciences (https://www.resdc.cn)
Spatial distribution of population	Spatial density data of the Chinese population with a spatial resolution of 1 km	2005, 2010	Resources and environment science and data center of Chinese Academy of Sciences (https://www.resdc.cn)
MCD12Q1	Spatial distribution data with a spatial resolution of 500 m	2010	MODIS product data official website (https://modis.gsfc.nasa.gov )
FFDAS	Annual carbon emissions spatial distribution data with a spatial resolution of 0.1° × 0.1°	2010	Ffdas data official website (https://ffdas.rc.nau.edu/)
ODIAC	Monthly carbon emissions spatial distribution data with a spatial resolution of 1 km × 1 km	2010	Odiac data official website (https://db.cger.nies.go.jp/dataset/ODIAC/)

#### Provincial scale carbon emissions data

Fossil energy consumption is the main source of carbon emissions. Eight major fossil fuels including coal, coke, crude oil, gasoline, kerosene, diesel oil, fuel oil, and natural gas were selected in this paper. Calculated as follows:

1 $$ SC = \mathop \sum \limits_{w = 1}^{W} E_{w} \times EC_{w} = E_{w} \times LC_{w} \times CC_{w} \times COF_{w} \times \frac{44}{{12}} $$

SC is CO_2_ emissions, E is the energy consumption, W is the type of fossil energy, EC is the carbon emissions coefficient, ALC is the average low calorific value (from the China Energy Statistics Yearbook), CC is the carbon content per unit calorific value, COF is the rate of oxidation of carbon and 44/12 is the coefficient of carbon to carbon dioxide. The calculation of the EC is based on the Guidelines for the Preparation of Provincial Greenhouse Gas Inventories (Fa Gai Ban Climate [2011] No.1041) and the China Energy Statistical Yearbook. CC and COF are derived from the Guidelines for Provincial Greenhouse Gas Inventories, and they are more applicable to the actual situation in China than the parameters provided by the IPCC^17^. LC is derived from the China Energy Statistical Yearbook. From this, the carbon emission coefficient of various energy sources in China can be calculated, as shown in Table 3. China only publishes complete provincial energy consumption data. The above formula was used to calculate the carbon emissions of 30 provinces in China except for Tibet, Hong Kong, Macao, and Taiwan in 2005 and 2010, with a unit of 10^4^ T.

**Table 3:** Carbon emission coefficients of various types of energy.

Energy type	Coal	Coke	Crude oil	Gasoline	Kerosene	Diesel	Fuel oil	Nature gas
LC (kJ/kg, kJ/m^3^)	20,908	28,435	41,816	43,070	43,070	42,652	41,816	38,931
CC (kg/GJ)	26.37	29.50	20.10	18.90	19.50	20.20	21.10	15.30
COF (%)	94%	93%	98%	98%	98%	98%	98%	99%
EC (kgCO_2_/kg, kgCO_2_/m^3^)	1.9003	2.8604	3.0202	2.9251	3.0179	3.0959	3.1705	2.1622

**Figure 7:**
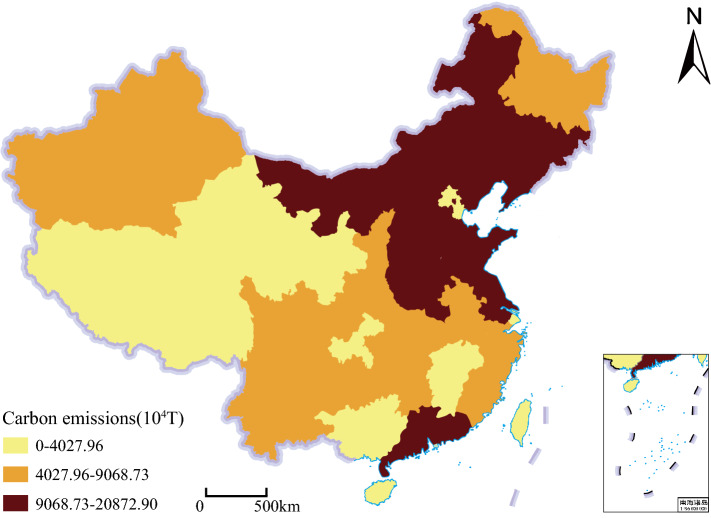
Distribution of provincial carbon emissions in 2010. This map is based on the standard map No. GS(2019) 1825 downloaded from the Ministry of Natural Resources Standard Map Service website. The base map is unchanged.

The distribution of provincial carbon emissions in the research area in 2010 is shown in Fig. 7.

#### Raster data

Nighttime lights data, population data, and GDP data were used as the driving factors for the spatialization of anthropogenic carbon emissions in this paper. Land cover data was selected as the comparison criteria for results.

The existing data sources of nighttime lights remote sensing data have been very diversified, such as DMSP-OLS, NPP-VIIRS, SAC-C-HSTC, and SAC-C-HSC. The data of 2010 and 2005 of the global NPP-VIIRS type nighttime lights data for cross-sensor calibration by Chen et al.^18^ was selected in this paper, with a spatial resolution of 500 m.

The national GDP data^19^ and population data^20^ were from the resources and environment science and data center. The unit of GDP data is 10000 yuan/km^2^, and the unit of population data is person/km^2^. The spatial resolution is 1 km.

TYPE 1 data of MCD12Q1 were used as land-use data (https://modis.gsfc.nasa.gov/data), which uses International Geosphere-Biosphere Program (IGBP) global vegetation classification method to classify vegetation into 17 land cover grades. Seventeen categories were reclassified into 7 land cover types in ArcGIS, including forest, shrubland, grasslands, water bodies, croplands, built-up and bare ground, with a spatial resolution of 500 m.

Raster data with different spatial resolutions cannot be used directly and requires some manipulation in ArcGIS software to produce raster data of the same size. Operations in ArcGIS software include setting a uniform coordinate system, using the Resample tool to unify the spatial resolution of the data, and using the Extract by Mask tool to clip the data to the study area. Since provincial-level data was required to complete the training of the model, various gridded data were zoned in ArcGIS to obtain provincial unit statistics for each driving factor.

#### Carbon emissions data

The Open-source Data Inventory for Anthropogenic CO_2_ (OIDAC) carbon emissions database^21,22^ and the Fossil Fuel Data Assimilation System (FFDAS) carbon emissions database^23^ were selected as the comparative data in this paper. OIDAC database spatialized carbon emissions by 1 km using power plant data and nighttime lights data, and the time scale is the month. The monthly data in the database was extracted through the mask to obtain the data of the required area, and the annual data were combined, and the unit was T/km^2^/Year. The spatial scale of the FFDAS database was 0.1°, and the unit was KgC/M^2^/Years. The data of the two carbon emissions databases were unified with the research scope in the data preprocessing stage.

### ZSSR

Low-resolution images can be restored to high-resolution images with this technique. The zero-shot super-resolution (ZSSR) model^16^ does not use external images as label data but instead enlarges and restores images by learning repetitive information inside the images. This feature makes it useful for data that does not have corresponding high- and low-resolution image pairs, such as carbon emissions. The ZSSR model first used the interpolation method to scale the input image to obtain multiple images, and then scaled the image again and performed rotation and mirror processing to obtain the corresponding "high and low resolution" image groups. The model’s processing process is shown in Fig. 2. The neural network used in the model was composed of 8 hidden layers, with 64 channels in each layer. Relu was used as the activation function and L1 was used as the loss function. The formula is as follows, where $y_{i}$ represents the target value and $f\left( {x_{i} } \right)$ represents the model output (estimated value). Set the initial learning rate to 0.001, and performed linear fitting on loss to obtain the slope and standard deviation. If the standard deviation is greater than the slope multiplied by some factor (1.5 by default), the learning rate is reduced by a factor of 10. Stop training until the learning rate is 10^−6^.

2 $$ loss\left( {x,y} \right) = \frac{1}{n}\mathop \sum \limits_{i = 1}^{n} |f\left( {x_{i} } \right) - y_{i} | $$

Although ZSSR can learn the local features of carbon emissions well, it needs at least one "low-resolution image" for its "scaling up feature learning”. Current carbon emissions data usually only have statistical data based on administrative units (provinces, counties, townships, etc.), and the distribution of carbon emissions within administrative regions is homogeneous. It is meaningless to use the ZSSR model to spatialize carbon emissions in this situation. Therefore, the lack of "low-resolution images" is the key problem that the ZSSR model needs to solve firstly in the study of carbon emissions spatialization.

### Two-stage model

**Figure 8:**
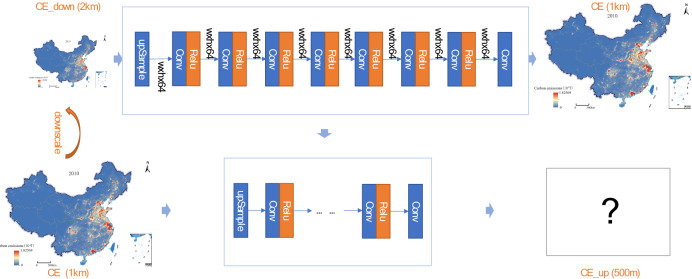
Zero-Shot Super-Resolution model. This map is based on the standard map No. GS(2019) 1823 downloaded from the Ministry of Natural Resources Standard Map Service website. The base map is unchanged.

The two-stage model aims to build a more comprehensive carbon emissions spatial model by introducing the ZSSR model to learn the local characteristics of carbon emissions. The accuracy of the ZSSR model is closely related to the quality of the input "low-resolution image". So, it is very important to build a "low-resolution image" that reflects the real spatial distribution of human carbon emissions. So it is very important to optimize the method of obtaining a "low-resolution image" and select the driving factors. The Pearson correlation coefficient method was used to analyze the correlation between carbon emissions and other independent variables. The closer the absolute value of the correlation coefficient is to 1, the stronger the correlation between the two variables. The nighttime lights data, population, and GDP data were the three data with the highest correlation coefficients, and the correlation analysis results were 0.84, 0.73, and 0.54. So they were selected as driving factors in this paper. In the selection of regression models, Support Vector Regression, Bayesian Linear Regression, Linear Regression, and ElasticNet Regression models were selected. The regression model can well learn the global characteristics of carbon emission distribution within an administrative region. Using drivers closely related to carbon emissions can ensure that the global features learned by the model are realistic. In the process of learning the global features, four regression models were used to learn the relationship between the provincial-level carbon emissions and the provincial-level driving factors first, and then the 1 km spatial result of carbon emissions was obtained by using the driving factor data, which was recorded as CE. Input CE into the ZSSR model, the model performed S-fold downsampling on CE to obtain CE_down, made CE as a label, and reconstructed CE_down into CE through Convolutional Neural Networks (CNN). After CNN learning was completed, input CE as a test image to CNN again to obtain the final 500 m carbon emission spatialization weight, named CE_up (Fig. 8). After the two-stage model, CE_up can learn not only the global characteristics of carbon emissions but also its local characteristics, which overcomes the defects of a single model in feature learning. In addition, it can break through the restriction of driving factors on the spatial resolution of regression results and improve the spatial resolution of carbon emissions to 500 m.

The result needs to be corrected with provincial carbon emission data to estimate the carbon emission value of each grid and form a national carbon emission spatial distribution map with zero error in provincial carbon emission (500 m spatial resolution). The specific formula is:

3 $$ CE_{c} = SC_{n} \times \left( {CW_{c} \div CW_{n} } \right) $$

where CE was the result of using provincial carbon emissions adjustment, SC represented provincial statistical carbon emissions data, CW represented the spatial weight result in ZSSR results, C represented a grid, and N represented a province.

### Experiment process

The research scale of this paper includes grid and provincial scales, and the spatial resolution of the grid scale is 500 m. This study used a two-stage model to complete the spatialization of carbon emissions, which was specifically divided into three basic steps: provincial scale regression model construction, "coarse" scale spatialization, and "fine" scale spatialization.

1. Construction of provincial scale regression model. The grid-scale driving factor data were zoning statistics to form provincial-level driving factors, and the provincial-level carbon emissions and driving factors were subjected to multi-model regression. Selected the regression model with the best results as the basic model for the next step.
2. "Coarse" scale spatialization. The grid-scale (1 km) driving factors were input into the optimal regression model to generate a 1 km carbon emissions spatialization result. The result was generated based on the global characteristics of carbon emissions. The result would be used as the "low-resolution image" input by the super-resolution model.
3. "Fine" scale spatialization. Input the "coarse" scale spatialization result into the ZSSR model to complete the spatialization of carbon emissions. The improvement of image resolution can be reflected in the doubling of the grid, which means the improvement of the spatial resolution. The ZSSR model can be used to obtain the spatial weight of carbon emissions with a spatial resolution of 500 m. By multiplying the provincial carbon emissions and spatial weights, the spatial results of carbon emissions with a spatial resolution of 500 m can be obtained.

**Figure 9:**
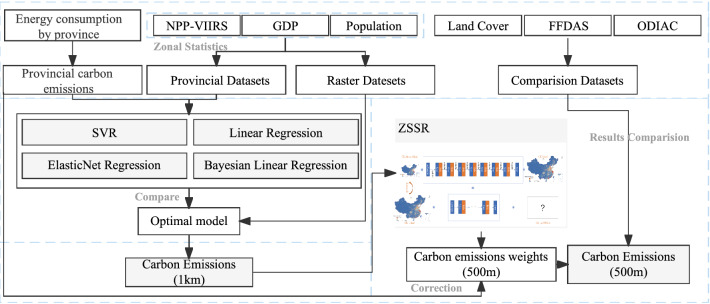
Experiment process.

The process can be summarized as using the traditional regression model to complete the mathematical relationship regression modeling, learning the overall characteristics of the spatial distribution, and using the ZSSR model to complete the local learning of the spatial distribution. Through these processes, the results can be more in line with the real situation (Fig. 9).

### Evaluation metrics

#### Statistical data evaluation indicators

Decision coefficient R^2^, mean absolute error (MAE) and root mean square error (RMSE) were used as indicators of the quantitative evaluation of the model. Taking the carbon emissions statistics of each province as the standard, the results of the model were quantitatively evaluated at the provincial level.

The value range of the decision coefficient R^2^ is [0,1]. The result is closer to 1, the better the result of the model. The formula is:

4 $$ R^{2} = 1 - \frac{{\mathop \sum \nolimits_{i} \left( {\widehat{{y_{i} }} - y_{i} } \right)^{2} }}{{\mathop \sum \nolimits_{i} \left( {\overline{{y_{i} }} - y_{i} } \right)^{2} }} $$

The average absolute error MAE records the average value of the prediction error, which is defined as follows:

5 $$ MAE = \frac{1}{m}\mathop \sum \limits_{i = 1}^{N} |h\left( {x_{i} } \right) - y_{i} | $$

The root means square error RMSE can more clearly show the degree of deviation between the predicted value and the real value. The larger the value, the greater the prediction error. The formula is:

6 $$ RMSE = \sqrt {\frac{1}{N}\mathop \sum \limits_{i = 1}^{n} \left( {Y_{i} - f\left( {x_{i} } \right)} \right)^{2} } $$

#### Spatial evaluation index

Most human social and economic activities are active in built-up land, which makes built-up land the most important source of human carbon emissions^24^. In China, the carbon emissions carried by built-up land have also exceeded 70% of the total carbon emissions^25^. Human social and economic activities in other land use types are not active, and carbon emissions are relatively small. By analyzing the spatial distribution relationship between land use data and carbon emissions data, the spatial accuracy of carbon emissions estimation can be accurately assessed^26^. The land use data include two spatial scales of 1 km and 500 m in this paper. The carbon emissions ratios of seven types of land use types were calculated respectively to analyze the spatial accuracy of carbon emissions estimation in this study. Compared the results with the carbon emission database FFDAS and ODIAC, analyzing the similarity of its carbon emission distribution. Compare the carbon emission proportion of construction land in ODIAC, SVR (Support Vector Regression) model and SVR-ZSSR (Support Vector Regression–Zero-Shot Super-Resolution) model, and quantitatively analyze the spatial accuracy according to the carbon emission proportion of built-up land.

## Data Availability

The datasets generated during and analyzed during the current study are available from the corresponding author on reasonable request.
